# Treatment of late left bronchopleural fistula after left pneumonectomy through right thoracic approach assisted by extracorporeal membrane oxygenation

**DOI:** 10.1186/s13019-024-02805-9

**Published:** 2024-05-31

**Authors:** Wenhao Li, Kejun Liu, Xiaozu Liao, Binfei Li, Yi Liang, Weizhao Huang

**Affiliations:** 1 Department of Cardiothoracic Surgery, Zhongshan City People’s Hospital, No.2 Sunwen Dong Road, Zhongshan, Guangdong Province China; 2 Department of Anesthesiology, Zhongshan City People’s Hospital, No.2 Sunwen Dong Road, Zhongshan, Guangdong Province China

**Keywords:** Bronchopleural fistula, Veno-venous extracorporeal membrane oxygenation, Pneumonectomy, Right thoracic approach

## Abstract

**Background:**

Bronchopleural fistula (BPF) is a rare but fatal complication after pneumonectomy. When a BPF occurs late (weeks to years postoperatively), direct resealing of the bronchial stump through the primary thoracic approach is challenging due to the risks of fibrothorax and injury to the pulmonary artery stump, and the surgical outcome is generally poor. Here, we report a case of late left BPF following left pneumonectomy successfully treated using a right thoracic approach assisted by extracorporeal membrane oxygenation (ECMO).

**Case presentation:**

We report the case of a 57-year-old male patient who underwent left lower and left upper lobectomy, respectively, for heterochronic double primary lung cancer. A left BPF was diagnosed at the 22nd month postoperatively, and conservative treatment was ineffective. Finally, the left BPF was cured by minimally invasive BPF closure surgery via the right thoracic approach with the support of veno-venous extracorporeal membrane oxygenation (VV-ECMO).

**Conclusions:**

Advanced BPF following left pneumonectomy can be achieved with an individualized treatment plan, and the right thoracic approach assisted by ECMO is a relatively simple and effective method, which could be considered as an additional treatment option for similar patients.

## Introduction

Bronchopleural fistula (BPF) is one of the most severe complications of pneumonectomy and is associated with high mortality [1–3]. Surgical intervention is generally required when the BPF is large or does not improve with conservative treatment. For early fistulas, the bronchial stump can be closed intraoperatively by direct suturing through the empty thoracic cavity, along with reinforcement using sutures. However, when a BPF occurs late (weeks to years postoperatively), direct resealing of the bronchial stump through the primary thoracic approach is challenging due to the risks of fibrothorax and injury to the pulmonary artery stump, and the surgical outcome is generally poor [4, 5]. Here, we report a case of late left BPF following left pneumonectomy successfully treated using a right thoracic approach assisted by extracorporeal membrane oxygenation (ECMO).

## Case presentation

**Fig. 1:**
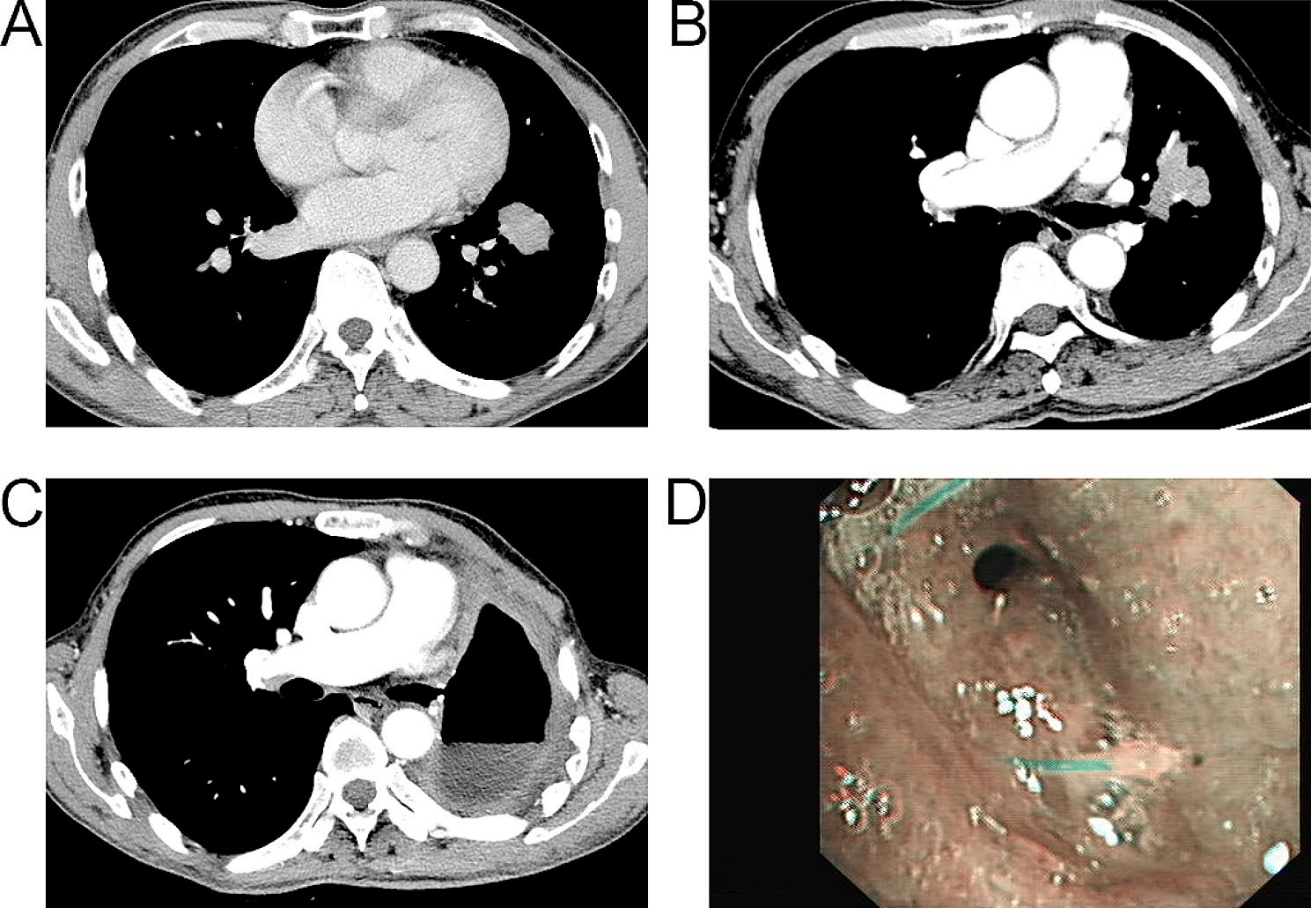
**A**. He was diagnosed with lung cancer in September 2018 following discovery of 27 mm×25 mm solid nodule in the left lower lung lobe. **B**. Lung cancer recurrence was later discovered in August 2021 following discovery of a 37 mm×26 mm mass adjacent to the left upper lung hilar. **C-D**. Chest CT and fibrobronchial examination revealed a small straight fistula at the left bronchial stump communicating with the left residual cavity

**Fig. 2:**
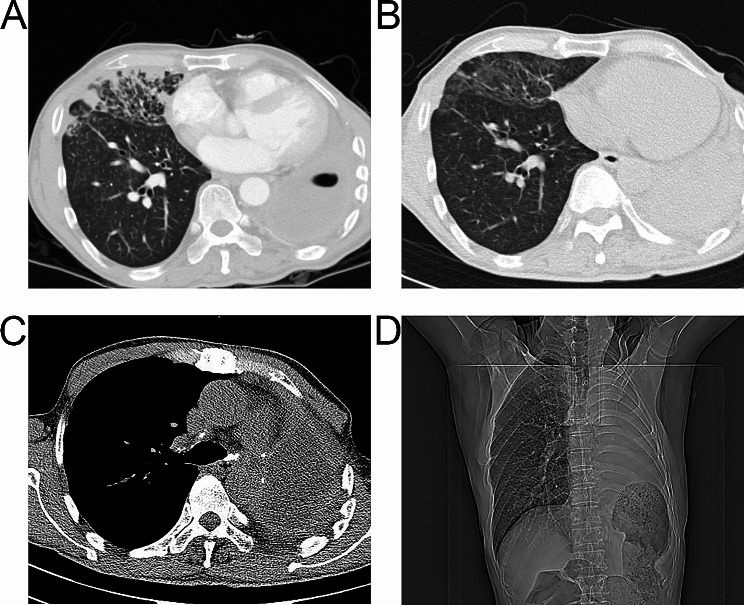
**A-B**. His right middle and lower pneumonia improved significantly compared with the previous period. No BPF could be seen on repeat chest CT within 3 months of postoperative follow-up. The residual cavity has been closed within 3 months of postoperative follow-up

A 57-year-old male was diagnosed with lung cancer in September 2018 following discovery of 27 mm×25 mm solid nodule in the anterior basal segment of the left lower lung lobe (Fig. 1A). He underwent left lower lobectomy, and postoperative pathological examination revealed moderately differentiated invasive adenocarcinoma without accompanying lymph node metastasis, resulting in a pathological diagnosis of pT1cN0M0 IA3 stage according to the 8th edition of the UICC TNM Classification. Lung cancer recurrence was later discovered in August 2021 following discovery of a 37 mm×26 mm mass adjacent to the left upper lung hilar (Fig. 1B). The patient was treated with left upper lobectomy, and postoperative pathological analysis revealed a low-differentiated squamous cell carcinoma without lymph node metastasis, pathologically diagnosed as pT2aN0M0 IB stage. The same patient was later admitted to the hospital with recurrent fever, cough, and bronchorrhea of tan fluid and purulent sputum for more than 1 month. He had previously been treated with various anti-infective regimens, with no symptom improvement. Chest computed tomography (CT) revealed postoperative changes after left pneumonectomy with left pleural thickening and encapsulated fluid pneumothorax, a small slit of about 3 mm in diameter locally visible in the left bronchial stump communicating with the left residual cavity (Fig. 1C), and right middle and lower pneumonia (Fig. 2A). Fiberoptic bronchus examination revealed a small fistula, approximately 3 mm in diameter, visible in the left bronchial stump (Fig. 1D). According to these findings, the patient was diagnosed with a left BPF.

**Fig. 3:**
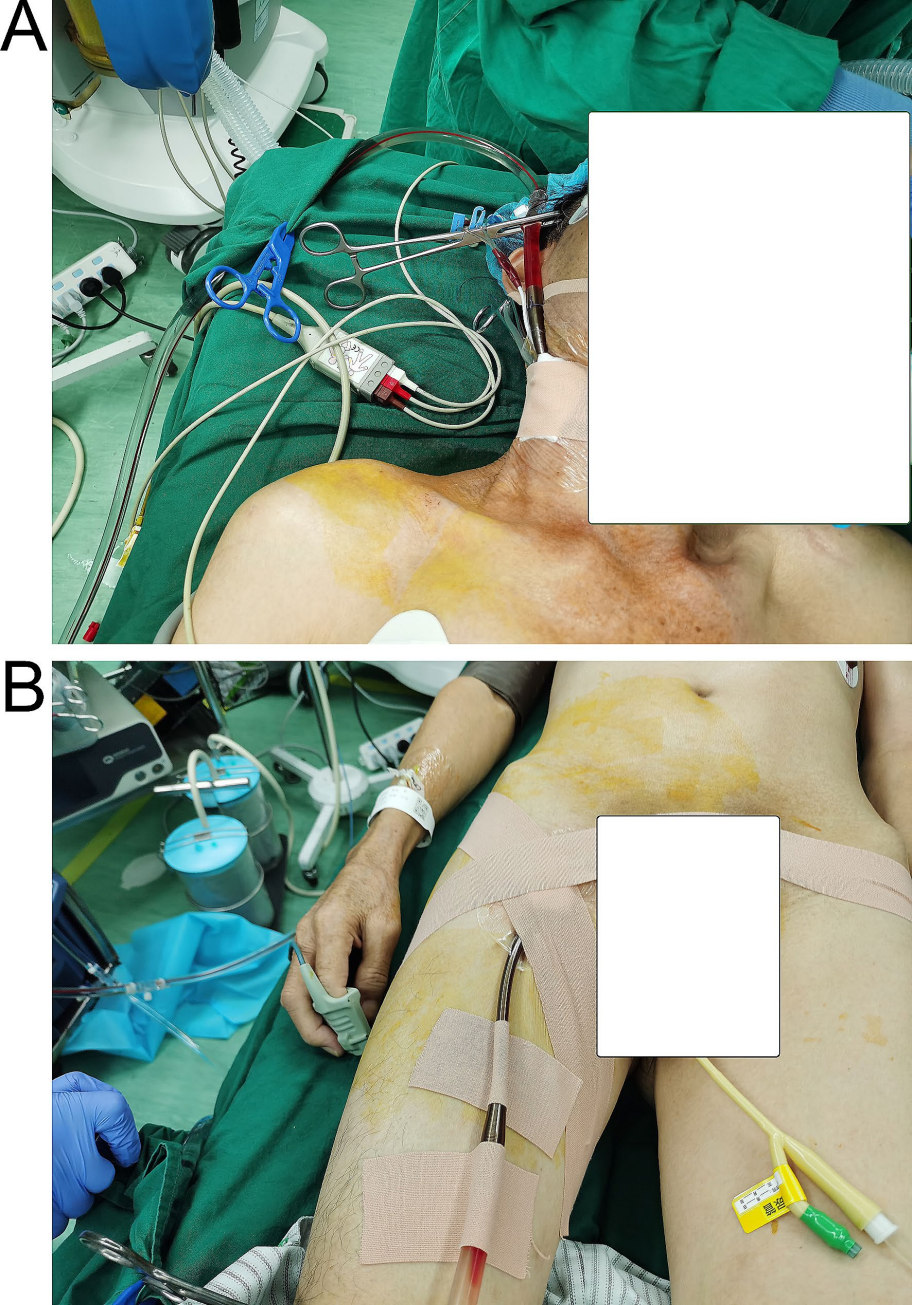
**A-B**. The cannulation strategy for VV-ECMO was to insert an outflow cannula via the right femoral vein and an inflow cannula via the right jugular vein

**Fig. 4:**
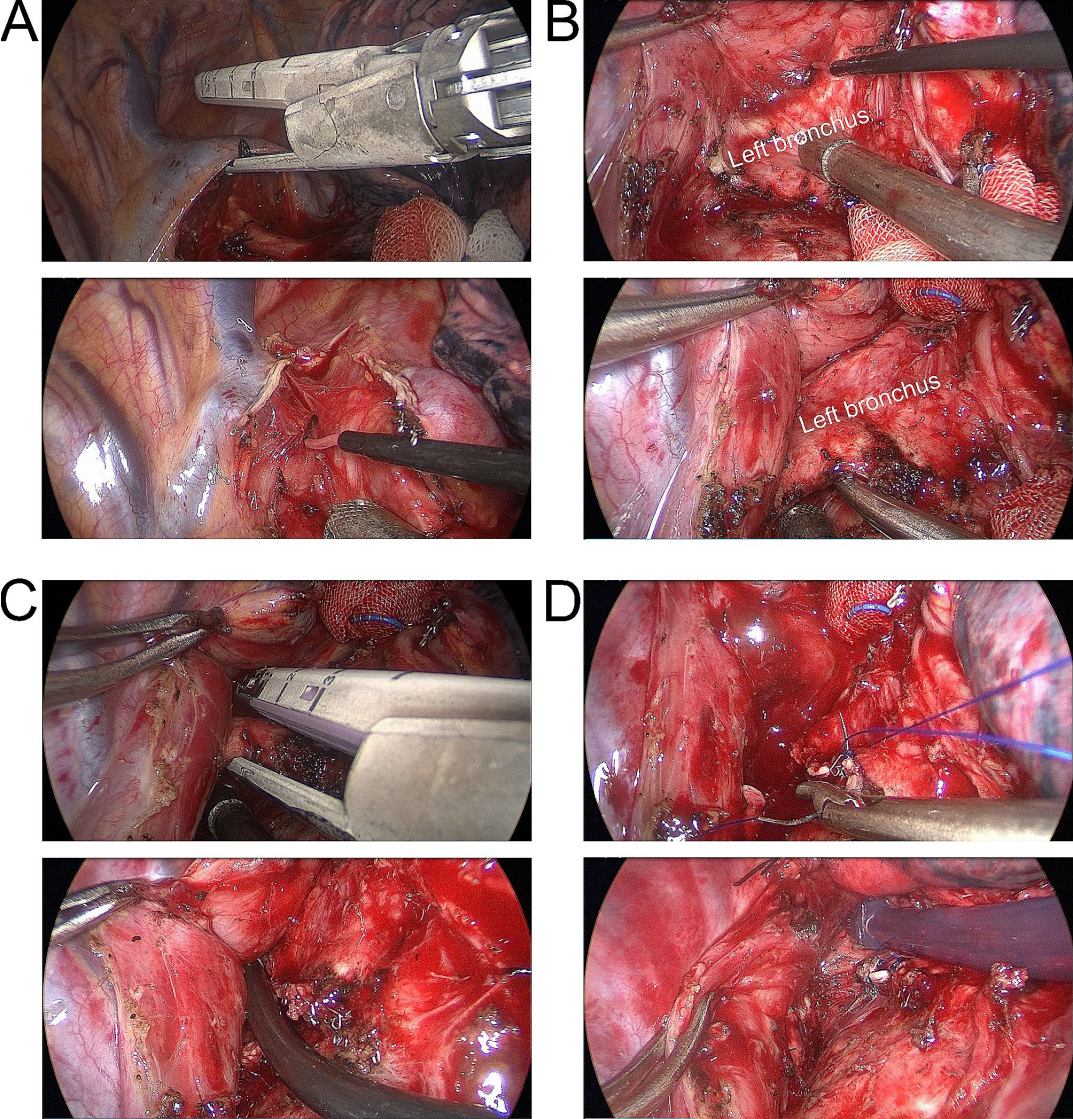
**A**. The arch of the singular vein was cut. **B**. The left main bronchus was dissected. **C-D**. The left main bronchus was disconnected with a suture to strengthen the severed end

Following 2 weeks of closed drainage of the left thoracic cavity and anti-infective treatment with a combination of imipenem-cilastatin sodium, linezolid, and voriconazole, the patient’s fever subsided, his cough and sputum symptoms improved, and his white blood cell count returned to normal. He was subsequently able to undergo BPF closure via a right-sided thoracic approach with the assistance of ECMO and thoracoscopy. This method requires oxygenation support with the assistance of veno-venous extracorporeal membrane oxygenation (VV-ECMO) due to the fact that the patient had only right lung tissue remaining. Our strategy was ultrasound-guided percutaneous cannulation, i.e., insertion of an outflow cannula via the right femoral vein and an inflow cannula via the right jugular vein (Fig. 3). A 4-cm incision was made in the fourth intercostal space between the right anterior axillary line and mid-axillary line to make an operation hole, and a 1-cm incision was made in the seventh intercostal space in the mid-axillary line to make an auxiliary hole to place the thoracoscope. Subsequently, the pleura of the right upper mediastinum was opened, and the arch of the singular vein was cut (Fig. 4A) to facilitate exposure of the operative area. The left main bronchus was dissected from the rondel to a length of about 3 cm (Fig. 4B), and then, the left main bronchus was disconnected using a stapler (Fig. 4C) with a suture to strengthen the severed end (Fig. 4D).

The operation was completed successfully, and ECMO was stopped immediately after the operation. The operative time was 136 min, intraoperative bleeding was approximately 105 mL, and VV-ECMO-assisted oxygenation support was provided for 115 min. In addition, antibiotic flushing and negative-pressure suction were applied after a closed drain was left in the left thoracic cavity, to control the pyothorax and promote closure of the residual cavity. The patient was able to get out of bed and move freely within 48 h after the operation, his right middle and lower pneumonia improved significantly compared with the previous period (Fig. 2B), the left thoracic residual cavity gradually narrowed, and the patient experienced no complications other than pain at the site of the surgical incision. The hospitalization time was 15 days. The patient was successfully discharged, and no BPF could be seen on repeat chest CT within 3 months of postoperative follow-up (Fig. 2C) and the residual cavity was closed (Fig. 2D).

## Discussion

Although BPF after pneumonectomy is rare, it remains a severe and often fatal complication. Early fistulas without pyothorax can typically be repaired immediately by thoracotomy with bronchial stump suture; however, in the present case, the interval between the left pneumonectomy and the diagnosis of the left main bronchial fistula was 22 months, and both a pyothorax and a fibrothorax had formed, making repair of the transthoracic bronchial stump highly complicated for multiple reasons. First, the septic environment created by a pyothorax prevents successful reconstruction of the bronchial stump; second, the post-pneumonectomy hiatus is typically heavily fibrotic, causing the hilar structures to adhere tightly to each other, while the tracheal fissures are located deep within the fibrous tissue covering the mediastinum, owing to which any attempts to dissect the bronchial stump may result in the risk of potentially-fatal hemorrhage due to injury to the pulmonary arterial stump. Therefore, direct reclosure of the bronchial stump via the primary thoracic approach is challenging. For this reason, we attempted to successfully treat the patient’s left BPF using closure of the left main bronchus through the right thoracic approach with the assistance of ECMO, achieving good results.

In the present case, the approach through the right chest was an alternative approach to bronchial stump dissection through a sterile space, which is technically simple and effective, as it avoids the infected area, scar tissue from the original surgical area, and the dissected bronchus and does not interfere with the fibrothorax that has already formed. However, after two successive lobectomies, the present case had the advantage of a mediastinal shift that was not particularly severe and a sufficiently long left main bronchial stump, providing feasibility for the successful application of this surgical approach. However, one disadvantage was that the abscess remained intact because closure of the BPF was not possible while dealing with the residual abscess space. Therefore, postoperative closed drainage and negative pressure suction of the left-sided abscess chest were needed to promote closure of the left-sided residual thoracic space.

Bronchial stump repair through a sternal and pericardial approach has previously been indicated as a viable method [4–6]. However, this approach is not suitable for patients with previous cardiac surgery [5] and requires prolonged hyperoxia and single-lung high-pressure ventilation in the healthy lung, which may readily exacerbate damage to the already infected healthy lung [7, 8]. The right thoracic approach allows for minimally invasive surgery without incision of the sternum and pericardium, resulting in a smaller incision and avoidance of cardiac influence; furthermore, due to the use of VV-ECMO support, there no need to ventilate the patient, as adequate oxygenation is provided during surgery, thereby avoiding damage to the healthy lung due to prolonged high-oxygen concentration and high-pressure ventilation.

VV-ECMO support provides oxygenation assurance and time for surgical treatment of left BPF via a right thoracic approach. Although the use of ECMO may increase the likelihood of intraoperative bleeding, based on our previous experience, we generally prescribe a short-term non-anticoagulant or hypo-anticoagulant for surgical patients undergoing ECMO-assisted procedures, and the ECMO is withdrawn as soon as ventilation is stabilized to avoid the risk of complications.

Bronchoscopic endoluminal occlusion technique may also be a viable treatment option. However, in clinical practice, the effect of endoluminal occlusion therapy varies greatly [9], on the one hand, due to the large heterogeneity among different patients, such as the location of bronchopleural fistula, the size of the fistula, as well as the general condition of the patient, whether or not the co-infection, etc.; on the other hand, the choice of the occlusion material also directly affects the effect of endoluminal occlusion therapy. In addition, BPF treated with bronchoscopic endoluminal occlusion may have some serious complications, including aggravation of infection, enlargement of the fistula opening due to the occlusion material, or even dislodgement of the occlusion material into the empty residual lumen, and so on. For BPF directly caused by tumors, treatment is mostly palliative. However, for BPF caused by non-tumor causes such as surgery, trauma, or infection, the principle of treatment is to try to obtain a radical cure. Therefore, surgical repair or suturing of the fistula is a more appropriate method of achieving a radical cure in patients such as the present case.

## Conclusions

In conclusion, advanced BPF following left pneumonectomy can be achieved with an individualized treatment plan, and the right thoracic approach assisted by ECMO is a relatively simple and effective method, which could be considered as an additional treatment option for similar patients.

## Data Availability

No datasets were generated or analysed during the current study.

## Abbreviations

VV-ECMO: Veno-venous extracorporeal membrane oxygenation
BPF: Bronchopleural fistula
CT: Computed tomography
